# Pseudo-Stener lesion due to proximal ulnar collateral ligament rupture: A case report

**DOI:** 10.1016/j.ijscr.2023.108141

**Published:** 2023-04-11

**Authors:** Griffin Lerman, Robert Bullock, Marc Trzeciak

**Affiliations:** Valley Consortium for Medical Education, Modesto, CA, USA

**Keywords:** Ulnar collateral ligament, Proximal rupture, gamekeeper's thumb, skier's thumb

## Abstract

**Introduction and importance:**

Ulnar collateral ligament (UCL) ruptures are a common injury of the thumb. The UCL will most commonly rupture at the distal insertion. It has been proposed that a partial or non-displaced tear can be managed non operatively. However, a complete rupture that occurs at the distal insertion will commonly not be able to heal non-operatively due to the interposition of the adductor aponeurosis. This clinical finding is known as a Stener lesion, first described by Bertil Stener in 1962.

**Case presentation:**

We present the case of a 63-year-old-woman with instability of the thumb, pain, and a small mass at the ulnar side of the metacarpal phalangeal joint (MCPJ).

**Clinical discussion:**

A Stener lesion mass is commonly palpated on the ulnar MCPJ due to the ligament being trapped proximally to the overlying aponeurosis. Our patient mistakenly presented with a Stener lesion that was intraoperatively discovered to be a mass of granulation tissue. This patient underwent repair of the UCL and was able to return to unrestricted daily activities after six weeks.

**Conclusion:**

This case highlights an unusual rupture pattern and shows the proper surgical techniques for repairing such an injury. It is imperative to restore joint stability to prevent decreased grip strength and early onset of osteoarthritis of the MCPJ.

**Level of evidence:**

Therapeutic Level 3B

## Highlights

• Gamekeeper's thumb and Skier's thumb injury presentation
• Stener lesion variant with a unique rupture pattern and similar presentation
• Proximal ulnar collateral ligament ruptures
• Surgical techniques for ulnar collateral ligament repair

## 1 Introduction

Ulnar collateral ligament (UCL) ruptures are a common injury, encompassing 86 % of injuries to the base of the thumb and affecting nearly 200,000 patients per year [1]. The UCL will rupture at the distal insertion or very rarely proximally or mid-substance. It has previously been proposed that in a partial or non-displaced tear, a thumb UCL sprain can typically be managed non operatively. A complete rupture that occurs at the distal insertion typically will not be able to heal non-operatively due to the interposition of the adductor aponeurosis which will cause a Stener lesion, first described by Bertil Stener in 1962 [2]. Rupture without a displaced UCL generally leads to complete recovery because there is no interposing structure between the healing ends of the UCL ligament. This case report has been reported in line with the SCARE criteria [3]. The patient provided consent for this case report and has remained anonymous throughout this study.

## 2 Presentation of case

A 63-year-old woman presented to the clinic with right thumb pain. The patient reported that 7 days prior, she “caught and twisted” her thumb on an object and had residual pain at the first MCPJ since then. The patient is a housekeeper and this injury had affected her capacity to work. Patient denied any past injuries to this area of her hand. Patient has had some pain reduction with acetaminophen. She had not undergone any trials of bracing, injections, or physical therapy prior to coming to the office. Past medical history of hypertension, hyperlipidemia and type two diabetes mellitus. Past surgical history is significant for a left ankle open reduction and internal fixation (ORIF) and right foot ORIF, bilateral carpal tunnel releases, and a cholecystectomy. At the time of presentation, she was taking metformin, losartan, atorvastatin, acetaminophen, and hydrochlorothiazide. The patient denied tobacco, alcohol, or illicit drug use. Family history was non-contributary, and she had no known drug or latex allergies.

On physical exam the skin of the first metacarpal of the right hand was intact, no erythema, swelling or bruising. On palpation, the patient reported pain on the dorsal and ulnar aspect of the thumb. A valgus stress test was performed and compared to the non-injured contralateral hand. A valgus force was directed at the right thumb, first in extension where the injured thumb deviated radially 50° (contralateral 20°) and then in flexion where there was 80° of deviation (contralateral 35°). There was no pain at the carpometacarpal joint or wrist. The patient was neurovascularly intact with brisk capillary refill. The remainder of the general exam was within normal limits.

**Fig. 1:**
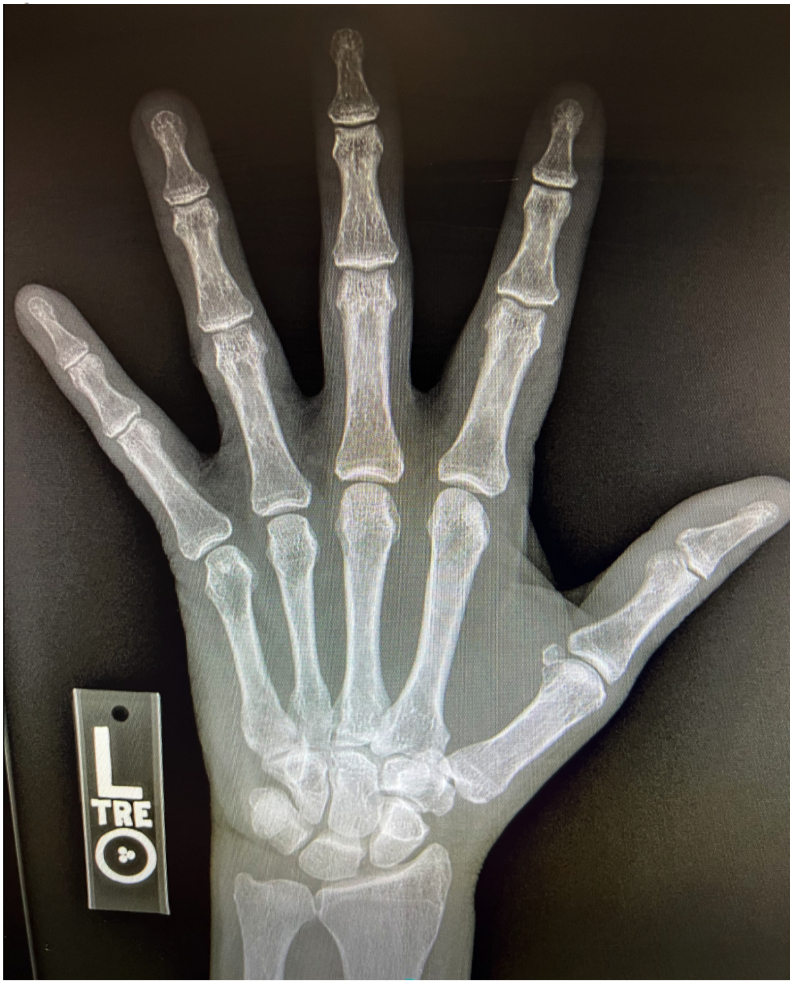
Radiographic image of hand.

**Fig. 2:**
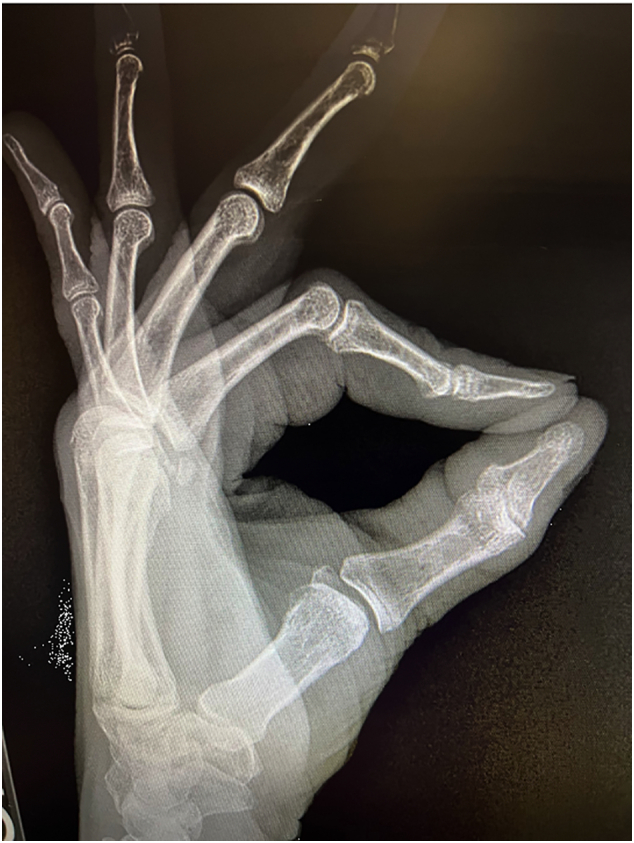
Radiographic image of hand.

**Fig. 3:**
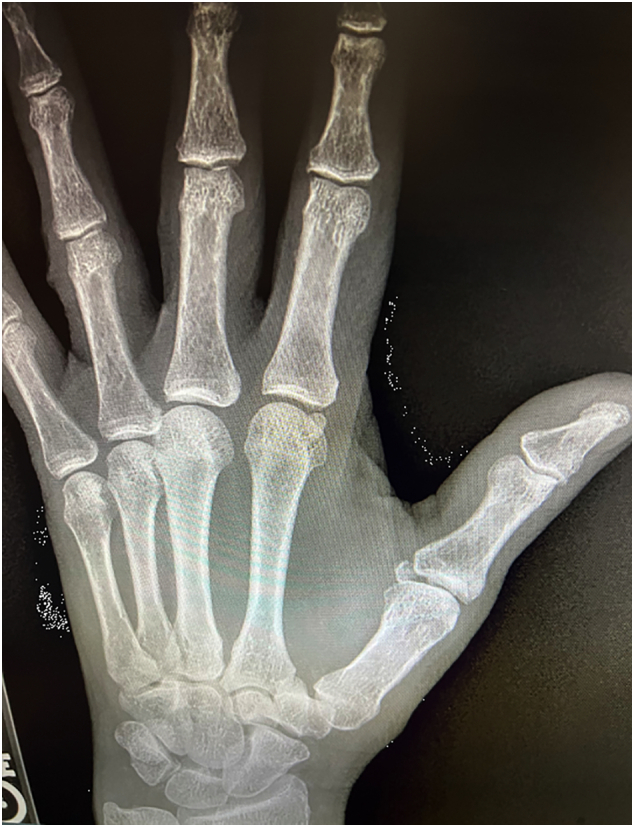
Radiographic image of hand.

X-Ray imaging from an outside facility (Fig. 1, Fig. 2, Fig. 3) demonstrated widening of the ulnar aspect of the 1st MCP joint in non-stress views. There were no bony avulsions, fractures, or dislocations. Based on patient history and compelling physical exam findings, the patient was diagnosed with a right thumb MCP UCL rupture. Patient elected to undergo operative intervention with thumb UCL Repair.

**Fig. 4:**
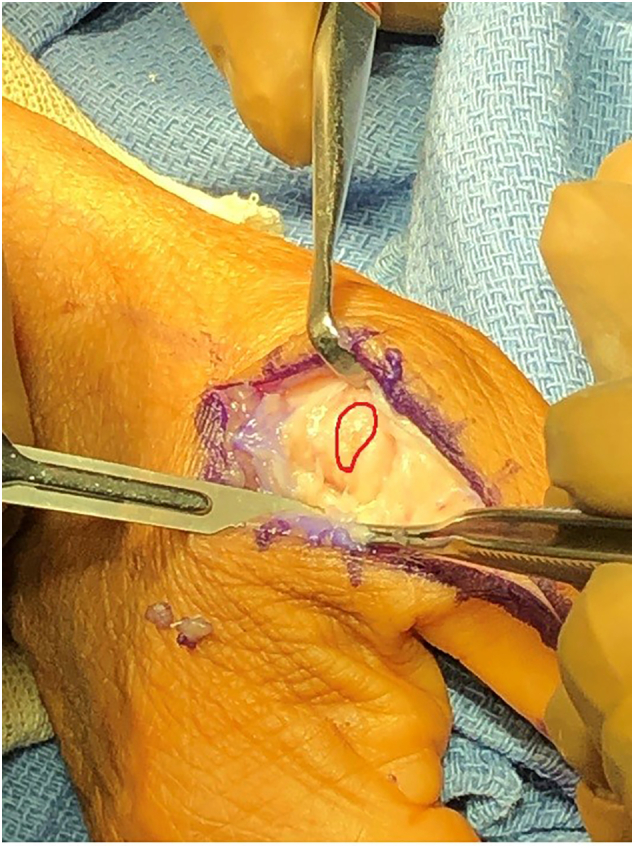
Proximal UCL avulsion.

**Fig. 5:**
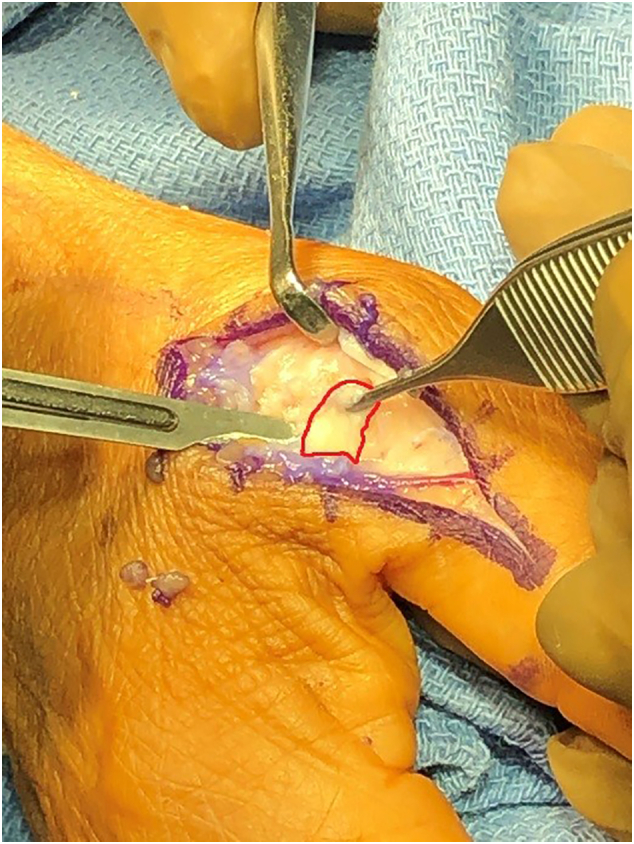
Proximal UCL avulsion.

The Surgery was performed forty-four days after the injury. The patient underwent general anesthesia, and the right upper extremity was prepped and draped in a sterile fashion. The thumb was examined, confirming the previous findings of laxity with a valgus stress test in both extension and flexion. A straight incision was made over the base of the proximal phalanx and head of the metacarpal. The dorsal branch of the digital nerve was identified and carefully retracted. The adductor aponeurosis was divided along its insertion on the dorsal extensor tendon expansion. The joint capsule was incised longitudinally and at this point the ulnar collateral ligament was exposed and avulsion at the proximal attachment was identified (Fig. 4, Fig. 5).

The UCL was repaired using Arthrex 2.5 mm PushLock® Knotless Suture Anchor and 2-0 fiber wire with a tapered needle. First a hole was drilled at a 90-degree angle to the cortex of the metacarpal head and the fiber wire was sutured through the tendon and passed through an eyelet. The eyelet was placed into the hole and the suture anchor mechanism was anchored by striking the head of the PushLock device. The anchor was tightened by hand and the excess sutures were cut flush. Using 3-0 Supramid nylon sutures, the volar plate was repaired and moving dorsally the adductor aponeurosis was reapproximated to the extensor apparatus and repaired as a separate layer. The thumb MCPJ was then pinned in extension using a 0.028-inch Kirschner wire, which remained in place for four weeks. The wound was closed using 4-0 nylon interrupted suture.

Postoperatively, the thumb was placed in a thumb spica splint for 48 h which was then exchanged for a cast for four weeks. After four weeks, the K-wire was removed, and the patient was able to resume light activities with a removable thumb spica brace. After six weeks the patient was unrestricted in her daily activities.

## 3 Discussion

An acute tear to the UCL is known colloquially as a Skier's thumb due to the mechanism of injury in the case of a skiing accident. The skier would fall and catch their thumb on a ski pole causing a traumatic valgus force on the MCPJ forcing it into hyperextension or hyperabduction. However, the most common mechanism for injury is a fall, usually at home, followed by sports, assaults, and direct blows [4]. In the case of chronic injuries (greater than six weeks), the injury is referred to as a Gamekeeper's thumb. First recognized by Campbell in 1955 to describe the UCL insufficiency Scottish gamekeepers from the maneuver repeatedly dispatching rabbits [4]. Although it is known that the UCL can tear distally, mid substance or proximally, nearly all cases in the literature report distal tears.

The UCL is 12–14 mm long and 5–8 mm wide. Its proximal attachment is the head of the metacarpal, and it attaches to the medial aspect and base of the proximal phalanx. The UCL can be subdivided into the proper collateral ligament and the accessory collateral ligament. The proper collateral ligament is more dorsal and is taut in flexion where it functions as a stabilizer of the MCPJ in flexion. Conversely, the accessory collateral ligament is more palmar and contributes to valgus stability in extension in conjunction with the volar plate [5], [6]. The dynamic stabilizers are extrinsic and intrinsic muscles as well as the adductor mechanism, which inserts into the extensor aponeurosis. The adductor pollicis tendon inserts partly through the ulnar sesamoid bone and partially through the extensor aponeurosis [2]. There are three grades of UCL tears, Grade 1 is microscopic tearing with no loss of ligament congruity, Grade 2 is a partial tear and Grade 3 (as in this case) is a complete rupture of the ligament [7]. In the case of a Stener lesion, the distal edge of the torn UCL becomes trapped superficial to the proximal edge of the adductor aponeurosis. It cannot heal without surgery because the aponeurosis prevents the ligament from meeting its distal attachment site on the proximal phalanx [2]. In Bertil Stener's original paper, among all his patients only a single patient presented with a proximal tear of the proper collateral ligament – which was incomplete, however it did have a concomitant complete tear of the accessory UCL [2].

In a study of 750 thumbs, Palmer et al. was able to conclude if there was greater than 35° of radial deviation with a valgus stress test in an otherwise intact joint, a physician can make the diagnosis of a complete rupture of the UCL [8]. Due to a wide range in variability in laxity, it can be more useful to compare to the contralateral thumb, where a difference in 15° or more of laxity with valgus stress can be indicative of a complete rupture [9]. It is essential to valgus test the thumb in flexion to isolate the proper collateral ligament, due to multiple stabilizing structures in extension. When testing the thumb in both flexion and extension the physician must counteract the rotational effects of the MCP. Based on physical exam findings such as excessive radial deviation, isolated or more notably greater than the contralateral hand as well as an absence of a firm endpoint can give an accurate clinical diagnosis of a complete rupture [10]. Although it is not diagnostic, in as few as a couple days after the injury, a palpable mass on the ulnar aspect of the metacarpal head may be present and is highly suggestive of a displaced ligament with granulation tissue [2], [11].

If physical exam maneuvers are limited by pain or swelling in the acute phase, various modalities have been shown to increase the efficacy of the clinical exam. Local anesthetic has been shown to increase the sensitivity of a valgus stress test [12]. Ultrasound can be used if available and various studies have shown to have a sensitivity ranging from 83 to 92 % and a positive predictive value (PPV) of 94–99 % [4], [14]. In many cases AP and Lateral radiographs are unable to discern bony avulsions of the UCL. However, our patient's history and physical exam was unmistakable for a UCL rupture and additional imaging was not necessary.

Conservative treatment is only reasonable if all stability tests have precluded the possibility of a complete UCL rupture [14]. In a Grade I or II lesion (not completely ruptured), casting can be sufficient, however, in the case of a complete rupture, even if the injury is minimally displaced, it has shown to be inadequate and resulted in longstanding pain and disability, even in the case of minimally displaced injuries [15], [16]. Ideal treatment should be done within the first 21 days of injury in which patients have reported good to excellent results in more than 90 % of the operations [5].

Ligamentous healing is a slow process and can take years. It is important to note that many other factors such as comorbid conditions (as in this patient who had type 2 diabetes) can impair healing quality. The thumb is inherently very mobile and the effect of increased mobility has been shown to cause distraction of the ligament ends [17]. In our patient, extensive granulation tissue formed at the proximal avulsion site creating a pseudo-Stener lesion that prevented ligamentous healing. As the gap between the two ends of the ligaments increase, the rate of scar maturation delays, and in the case of this lesion, the ligament had formed adhesions to the local structures and the granulation tissue did not appear to advance towards its attachment site, thus preventing healing [18].

Literature has shown that delays in treatment correlate with worse patient outcomes. Chuter et al. proposed that repair of the acute UCL rupture (within 6 weeks) is ideal and an easier procedure whereas delayed treatment usually required a reconstruction [13]. Despite different requirements for treatment based on the acuity of the injury, a review by Samora et al. has proposed that regardless of repair or reconstruction patient outcomes are satisfactory after 2 years [19]. It has been proposed that a UCL ligament can heal without operative management if the UCL is not displaced [5]. Some studies have shown a failure rate of 50 % with closed treatment [1].

## 4 Conclusion

If the UCL displaces and forms a Stener lesion, there will likely be no ability to heal due to the ligaments losing contact to their attachment. In our case, the excessive granulation tissue formed a pseudo-Stener lesion that precluded ligamentous healing. We propose that judicious care should be taken to undergo rapid repair of a torn UCL regardless of its location because scar tissue can interdigitate and form a pseudo-Stener lesion even with a proximal avulsion. Our patient demonstrated an unusual tear location and a successful treatment protocol. This case highlights that early diagnosis is key, and fortunately this patient was successfully treated with a ligament repair.

## Patient's consent

Written informed consent was obtained from the patient for publication of this case report and accompanying images. A copy of the written consent is available for review by the Editor-in-Chief of this journal on request.

## Ethical approval

Ethical approval was provided by the author's institution.

## Funding

N/A.

## Guarantor

Trzeciak, Marc DO.

## Research registration number

1. Name of the registry: N/A
2. Unique identifying number or registration ID: N/A
3. Hyperlink to your specific registration: N/A.

## CRediT authorship contribution statement

Lerman, Griffin DO – corresponding author, wrote the paper, assisted with case
Bullock, Robert DO – Wrote operative report and assisted with case
Trzeciak, Marc DO – Attending.

## Conflicts of interest

N/A
